# Nox1-based NADPH oxidase regulates the Par protein complex activity to control cell polarization

**DOI:** 10.3389/fcell.2023.1231489

**Published:** 2023-08-11

**Authors:** Alejandra Valdivia, Charity Duran, Mingyoung Lee, Holly C. Williams, Moo-Yeol Lee, Alejandra San Martin

**Affiliations:** ^1^ Division of Cardiology, Department of Medicine, School of Medicine, Emory University, Atlanta, GA, United States; ^2^ BK21 FOUR Team and Integrated Research Institute for Drug Development, College of Pharmacy, Dongguk University, Goyang, Republic of Korea; ^3^ Institute of Biomedical Sciences, Faculty of Medicine and Faculty of Life Science, Universidad Andres Bello, Santiago, Chile

**Keywords:** Nox1, Polarity, Par3, migration, PP2A

## Abstract

Cell migration is essential for many biological and pathological processes. Establishing cell polarity with a trailing edge and forming a single lamellipodium at the leading edge of the cell is crucial for efficient directional cell migration and is a hallmark of mesenchymal cell motility. Lamellipodia formation is regulated by spatial-temporal activation of the small GTPases Rac and Cdc42 at the front edge, and RhoA at the rear end. At a molecular level, partitioning-defective (Par) protein complex comprising Par3, Par6, and atypical Protein Kinase (aPKC isoforms ζ and λ/ι) regulates front-rear axis polarization. At the front edge, integrin clustering activates Cdc42, prompting the formation of Par3/Par6/aPKC complexes to modulate MTOC positioning and microtubule stabilization. Consequently, the Par3/Par6/aPKC complex recruits Rac1-GEF Tiam to activate Rac1, leading to lamellipodium formation. At the rear end, RhoA-ROCK phosphorylates Par3 disrupting its interaction with Tiam and inactivating Rac1. RhoA activity at the rear end allows the formation of focal adhesions and stress fibers necessary to generate the traction forces that allow cell movement. Nox1-based NADPH oxidase is necessary for PDGF-induced migration *in vitro* and *in vivo* for many cell types, including fibroblasts and smooth muscle cells. Here, we report that Nox1-deficient cells failed to acquire a normal front-to-rear polarity, polarize MTOC, and form a single lamellipodium. Instead, these cells form multiple protrusions that accumulate Par3 and active Tiam. The exogenous addition of H_2_O_2_ rescues this phenotype and is associated with the hyperactivation of Par3, Tiam, and Rac1. Mechanistically, Nox1 deficiency induces the inactivation of PP2A phosphatase, leading to increased activation of aPKC. These results were validated in Nox1^y/-^ primary mouse aortic smooth muscle cells (MASMCs), which also showed PP2A inactivation after PDGF-BB stimulation consistent with exacerbated activation of aPKC. Moreover, we evaluated the physiological relevance of this signaling pathway using a femoral artery wire injury model to generate neointimal hyperplasia. Nox1^y/-^ mice showed increased staining for the inactive form of PP2A and increased signal for active aPKC, suggesting that PP2A and aPKC activities might contribute to reducing neointima formation observed in the arteries of Nox1^y/-^ mice.

## 1 Introduction

Cell migration is a fundamental process for embryological development and tissue repair, while aberrant migration participates in diseases such as cancer, atherosclerosis, and restenosis (Schwartz, 1997).

Different cells in higher organisms display various migration modes depending on tissue environment, genetic background, and extracellular stimuli. Mesenchymal migration types, similar to those observed in fibroblasts and vascular smooth muscle cells, are characterized by strong adhesion to the substrate and cytoskeleton-mediated cell polarization.

In order to polarize, the cell relies upon specialized signaling domains to define the direction of eventual movement. During this process, the plasma membrane extends towards the stimulus in the form of a lamellipodium (Lauffenburger and Horwitz, 1996; Carpenter, 2000; Ballestrem et al., 2001; Small et al., 2002; Raftopoulou and Hall, 2004). Lamellipodia formation is driven by actin dynamics regulated by various signaling pathways (Petrie et al., 2009). Notably, the spatial-temporal activation of the small GTPases Rac and Cdc42 at the front and RhoA at the rear end leads to directional migration (Nobes and Hall, 1995; Raftopoulou and Hall, 2004).

At the molecular level, cell polarity and directional cell motility are regulated by a group of highly evolutionarily conserved proteins named partitioning-defective (Par) complex, consisting of Par3 (Pard3, aka Bazooka in *Drosophila*), Par6, and atypical Protein Kinase C (aPKC comprising isoforms ζ and λ/ι). The Par complex signals through Rho GTPases to control the basal-apical polarity of epithelial cells, asymmetric cell division, and the front-rear axis polarization during directional and persistent cell migration (Goldstein and Macara, 2007; Etienne-Manneville, 2008; Petrie et al., 2009; Chen and Zhang, 2013).

The role of Par3 in directional cell migration relies on its spatially regulated interaction with PAR6-aPKC during the establishment of front-rear cell polarity. Integrin clustering activates Cdc42 at the leading edge triggering the formation of Par3/Par6/aPKC complexes, which modulates MTOC positioning through dynein/dynactin and microtubule stabilization (Etienne-Manneville and Hall, 2001; Etienne-Manneville et al., 2005; Gundersen et al., 2005; Jaffe and Hall, 2005; Vicente-Manzanares et al., 2005; Osmani et al., 2006; Pegtel et al., 2007). Additionally, when the Par3/Par6/aPKC complex is formed, the Rac1-GEF Tiam (T-lymphoma invasion and metastasis-inducing protein) is recruited, leading to Rac activation and lamellipodium formation (Etienne-Manneville and Hall, 2001; Nishimura et al., 2005; Pegtel et al., 2007). At the rear end, the RhoA effector ROCK phosphorylates Par3 and disrupts its interaction with Tiam inactivating Rac. This allows focal adhesions and stress fibers to lead to the traction force that allows cell movement (Nakayama et al., 2008).

The role of reactive oxygen species (ROS), such as superoxide (O_2_•-) and hydrogen peroxide (H_2_O_2_) as signaling molecules, is widely accepted (Griendling et al., 2000; Lennicke and Cocheme, 2021). Furthermore, redox-dependent signaling is required for agonist-induced cytoskeleton reorganization and migration (Sundaresan et al., 1995; Nishio and Watanabe, 1997; Wang et al., 2001). However, the molecular targets of redox-sensitive signaling during migration are not fully elucidated.

NADPH oxidases are a primary enzymatic source of ROS in various biological systems. NADPH oxidases are multi-subunit enzymes whose superoxide-producing catalytic subunit consists of one of the Nox proteins and several structural and regulatory proteins (Lassegue et al., 2012). Nox1 activity is essential in fibroblast and smooth muscle cell migration (Lassegue et al., 2001; San Martin et al., 2007; Schroder et al., 2007; Lee et al., 2009; Maheswaranathan et al., 2011; Jagadeesha et al., 2012; Maheswaranathan et al., 2020). We have previously shown that the Nox1-based NADPH oxidase is necessary for PDGF-induced migration *in vitro* and *in vivo* (Lee et al., 2009).

This work aims to gain insight into the distinct molecular mechanisms by which Nox1 mediates the early cellular events leading to directional migration. Our results show that after PDGF stimulation, Nox1 deficient cells lose the ability to acquire normal front-rear polarity and to form a single lamellipodium. Instead, these cells form multiple protrusions that resemble little lamellipodia that amass Par3 protein and active Tiam.

Mechanistically, this effect is mediated by the Nox1-dependent mislocation and inactivation of the phosphatase PP2A leading to increase phosphorylation and aberrant activation of the Par3/aPKC/Tiam/Rac polarity complex.

## 2 Material and methods

### 2.1 Animals

Nox1^y/-^ mice were generated by Dr. K. H. Krause (Gavazzi et al., 2006) and backcrossed onto a C57Bl/6 background. The Institutional Animal Care and Use Committee of Emory University School of Medicine approved the animal protocol used in this study.

### 2.2 Mouse femoral artery injury model

Transluminal mechanical injury of bilateral femoral arteries was induced by introducing a large wire, as previously reported (Sata et al., 2000; Lee et al., 2009). At 21 days, the mice were sacrificed and pressure-perfused at 100 mmHg with 0.9% sodium chloride, followed by pressure fixation with 10% formalin. Arteries were then carefully excised, embedded in paraffin, and processed for histological analysis.

### 2.3 Histological analysis

Histology sections from WT and Nox1^y/-^ mice femoral arteries subjected to wire-induced injury were kindly provided by Dr. Kathy Griendling (Lee et al., 2009). Antigen retrieval was heat-induced in citrate buffer. Immunohistochemistry followed by DAB staining was performed using antibodies against phospho-Y307-PP2A-CA (Santa Cruz) and phospho-T410/403-PKCζ/λ (Cell Signaling) to determine the levels of inactivation of PP2A-CA and activation of aPKC, respectively. Images of the whole femoral artery were captured using a NanoZoomer SQ (Hamamatsu) using the 40X scanning mode. Approximately 3-5 femoral artery sequential slices were evaluated per animal, and a total of 5 animals were used per phenotype. In addition, the relative intensity of DAB staining was assessed using ImageJ.

### 2.4 Preparation of mouse embryonic fibroblast and mouse aortic smooth muscle cells

Mouse embryonic fibroblasts (MEFs) were prepared from wild-type (WT) and Nox1^y/-^ mouse embryos as described before (Brown et al., 2014). Briefly, E13.5 embryos were isolated with their yolk sacs and dissected to remove and discard the head and internal organs. The yolk sac was removed and retained for genotyping. The dissected embryo was passed through an 18G needle and subsequently plated on gelatin-coated dishes in Dulbecco’s modified Eagle’s medium (DMEM) high glucose (Sigma) supplemented with 15% fetal bovine serum (Sigma). After two passages, MEFs were immortalized by expression of the SV40 large T-antigen (Addgene plasmid 13,970). MEFs were subcultured at a 1:10 ratio for nine passages upon reaching confluence. Immortalized MEFs were grown in DMEM high glucose supplemented with 10% fetal bovine serum (Atlanta Biological), 1% penicillin-streptomycin, and 1% Glutamax (Gibco) and used for experiments for additional 15 passages.

Mouse aortic smooth muscle cells (MASMCs) were isolated from wild-type (WT) and Nox1^y/-^ mice by enzymatic dissociation (Fernandez et al., 2015). Cells were cultured in Dulbecco’s Modified Eagle’s Medium (DMEM) supplemented with 10% fetal bovine serum), 1% penicillin-streptomycin, and 1% Glutamax (Gibco). All cultures were used between passages 6 and 10 for experiments. Cultures at 70%–80% confluence were made quiescent by incubation in serum-free media for 16 h before the experiments.

All the cell lines were grown in a 5% CO2 atmosphere at 37 °C.

### 2.5 Antibodies and reagents

Antibodies used in these experiments are described in Table 1. Rabbit polyclonal anti-Par3 antibodies were used for immunoprecipitation (Proteintech) and immunofluorescence (Millipore). Rabbit anti-pY307PP2A-CA (Upstate/Millipore and Santa Cruz), Rabbit anti-PKCζ/ι, anti-Cortactin, Rabbit anti-α/β Tubulin (Cell Signaling), rabbit anti-Tiam1, mouse PKCζ/ι (Santa Cruz), mouse anti-GST (Proteintech), anti-Actin (Sigma), anti-FITC (Invitrogen), PDGF-R (EMD Millipore). Primary antibodies were used in 1/1,000 dilution for immunoblotting and 1:200 for immunofluorescence and immunohistochemistry experiments. All secondary antibodies conjugated to HRP or to Alexa fluorophores were from Jackson ImmunoResearch and were used 1/3,000 for immunoblotting and 1/200 for immunofluorescence and immunohistochemistry experiments. Okadaic Acid (Cayman Chemical Company) was used at 1 μM for 30 min before PDGF-BB (R&D systems) stimulation. EZ-Link Sulfo-NHS-SS-Biotin (Life Technologies) and 5-Iodoacetoamido-fluorescein (Sigma) were used according to manufacture protocols.

**TABLE 1:** Antibodies used in this manuscript.

Antibody	Company	Catalog #
p-Y307-PP2A-CA	Santa Cruz	sc-12615
p-Y307-PP2A-CA	Upstate/Millipore	05–547 (clone 4B10)
p-T410/403-PKCζ/λ	Cell Signaling	9378S
p-T410/403-PKCζ/λ	Abcam	ab76129
PKCζ/ι	Santa Cruz Biotechnology	sc-17781
PKCζ/ι	Santa Cruz Biotechnology	sc-216
PKCζ/ι	Santa Cruz Biotechnology	sc-7262
Cortactin	Cell Signaling	3503S
α/β-Tubulin	Cell Signaling	2148S
Tiam1	Santa Cruz	sc-872
GST	Proteintech	66001-1-Ig
β-Actin	Sigma	A5441
FITC	Invitrogen	71–1900
PDGFRβ	EMD Millipore	05-825R
Par3	EMD Millipore	07–330
Par3	Proteintech	11085-1-AP
Control non-immune IgG	Santa Cruz	sc-2025

### 2.6 siRNA and adenoviral infection

FlexiTube siRNA for mouse PP2A-CA sequences #4 and #7 were obtained from (Qiagen). Cells were seeded on coverslips and, after 16 h, were co-transfected with siGlo RNAi (GE Dharmacon) and siCtrl AllStars (Qiagen) or siRNA against PP2A-CA, using Lipofectamine RNAiMAX reagent (Thermo Fischer Scientific) according to the manufacturer recommendations. After 24 h of transfection, cells were serum starved and processed for immunofluorescence.

pAdEasy vector, which contains green fluorescent protein (GFP), was used to prepare viruses without an insert (pAdEasy-Ctrl) or hemagglutinin (HA)-tagged Nox1 (pAdEasy-Nox1-HA) as described before (Hanna et al., 2004). Cells were infected with the adenoviruses for 16 h in complete media. Subsequently, cells were trypsinized and seeded on coverslips for immunofluorescence assays.

### 2.7 Phospho-protein analysis

Cells were seeded on 15 cm dishes, allowed to attach for 5-6 h, and then serum-starved overnight. The following day, the media was refreshed, and cells were stimulated with PDGF 10 ng/mL for 30 min. We analyzed phosphorylated proteins by affinity chromatography using PhosphoProtein Purification Kit (Qiagen) according to the manufacturer’s indications. Purified phosphoproteins were resolved by electrophoresis and blotted for Par3, Cortactin, and Actin.

In other experiments, to analyze phosphorylation of pY307-PP2A-CA and pThr410/403-PKCζ/ι, cells were lysed in boiling-2x Lamelli buffer, sonicated, and immediately subjected to electrophoresis.

### 2.8 Rac1 activity assay

Activation of Rac1 was determined by G-LISA kit (Cytoskeleton). Briefly, cells were seeded on 6-well plates, serum-starved overnight, and stimulated with PDGF-BB 10 ng/mL for the indicated times. Protocol was followed according to the manufacturer’s recommendations. Results were the average of three independent experiments in which each condition was performed in triplicate.

### 2.9 Immunofluorescence

Cells were seeded on acid-washed coverslips coated with Collagen type I (Corning). After cell attachment (5–6 h), cells were serum starved for 16 h. The media was refreshed the following day. Cells were stimulated with PDGF-BB 10 ng/mL for 30 min. Media was removed, cells were immediately fixed in 4% Paraformaldehyde for 10 min, followed by permeabilization with 0.1% Triton X-100 for 10 min. Subsequently, coverslips were blocked for 10 min with 2% IgG-free BSA (Jackson Immuno Research), 0.1% Fish gelatin (Sigma) in UB buffer (50 mM Tris-HCl, pH 7.6; 0.15 N NaCl and 0.1% sodium azide). Primary antibodies were added for 1 h at room temperature and washed three times for 5 min each time with UB-0.1% Tween-20. Cells were blocked again as described above and then incubated with secondary antibodies conjugated to fluorophores, phalloidin, and DAPI for 1 h at room temperature. Coverslips were washed three times for 5 min in UB-0.1% Tween-20 and mounted in Mowiol solution. In some experiments, cells were seeded, and the next day they were co-transfected with siGlo RNAi and siRNA Control or siRNA PP2A-CA. During the Nox1 rescue experiments, cells were infected with the control virus (pAdEasy-Ctrl) or Nox1-HA (pAdEasy-Nox1-HA), and 24 h later, they were trypsinized and seeded on coverslips and processed as described above.

Lamellipodia formation was evaluated by double staining of cortactin and phalloidin using a Zeiss LSM 510 META or an LSM 800 Airyscan Laser Scanning Confocal Microscopes (Plan-Apo 63x NA 1.4 oil or Plan-Apo 20 × 0.8 NA). Pictures and analysis were performed blinded. Results are mean ± SEM of at least three independent experiments in which 10–100 cells were evaluated per condition.

### 2.10 Far immunofluorescence for active Tiam

MEFs were seeded on coverslips. After 24 h, cells were serum starved for 2 h and stimulated with 10 ng/mL PDGF-BB for 30 min. Then, cells were processed for a modified protocol of Far-immunofluorescence as previously described (Bustos et al., 2012; Pelletan et al., 2015; Valdivia et al., 2020). Briefly, cells were fixed, permeabilized, and blocked as described for immunofluorescence above. Subsequently, samples were incubated with 25 μg GST-Rac1G15A purified recombinant protein for 60 min and washed. Then, samples were blocked and incubated with anti-Tiam (Santa Cruz) and anti-GST antibodies for 60 min and washed. Coverslips were then blocked and incubated with anti-mouse AlexaFluor 568 and anti-rabbit AlexaFluor 633 (Jackson Immunological). Purified recombinant GST was used as a negative control. Both GST and GST-Rac1G15A were prepared as previously described (Garcia-Mata et al., 2006). Images were captured with LSM 800 Airyscan Laser Scanning Confocal Microscopes (Plan-Apo 63x NA 1.4 oil). Pictures and analysis were performed blinded. Results are mean ± SEM of at least three independent experiments in which 5–7 cells were evaluated in each condition.

Colocalization was quantified using Pearson’s R coefficients between two different channels within specific ROI located at the membrane of lamellipodium and lamellipodia-like protrusions using the Coloc2 macro in ImageJ. Values between 0.7 and 1 were considered true colocalization.

### 2.11 Migration assays

Migration was measured using a Boyden chamber. Cells were serum starved and allowed to migrate towards 10 ng/mL PDGF for 3 h. Migrated cells were stained with DAPI. Four random fields were visualized using Plan-Neo 20 × 0.5 NA in a Zeiss Axioskop2 wide-field microscope and quantified with ImageJ.

WT and Nox1^y/-^ MEFs were seeded in 2 well silicone inserts (Ibidi) for the wound healing assays and let grow to confluency overnight. Cells were serum starved for 2 h, and the external part of the dish was filled with starvation media containing 10 ng/mL PDGF-BB. Then, the silicone inserts were carefully removed, allowing the cells to migrate to the empty area for 30 min. In some experiments, cells were fixed and processed for immunofluorescence, as described above. The staining was performed using γ-Tubulin (Sigma) as a marker of MTOC and GM130 (ECM Bioscience) as a marker of Golgi. Cells were considered polarized when Golgi or MTOC were positioned within an angle of 120° in front of the nucleus facing the wound area and as not polarized when the signal was behind this angle or on top of the nucleus.

### 2.12 Statistical analysis

Data are expressed as the mean ± standard error of mean (mean ± SEM from at least three independent experiments (n = 3). In every independent experiment, for each experimental conditions we evaluated either 5–10 random field of view and counted 10–700 cells depending on the magnification used to capture the images. Data were compared using t-student test, 1-way or 2-way ANOVA analysis, accordingly. Significant differences were established at *p* < 0.05.

## 3 Results

### 3.1 Nox1-derived ROS regulate cell polarization

Similar to what has been reported in a variety of cell types (Schroder et al., 2007; Sadok et al., 2008; Lee et al., 2009; Shinohara et al., 2010; Khoshnevisan et al., 2020), Nox1 deficient (Nox1^y/-^) MEFs have impaired PDGF-induced migration (Supplementary Figure S1) despite having no differences in PDGFR levels at the plasma membrane when compared to control cells (Supplementary Figure S2).

**FIGURE 1:**
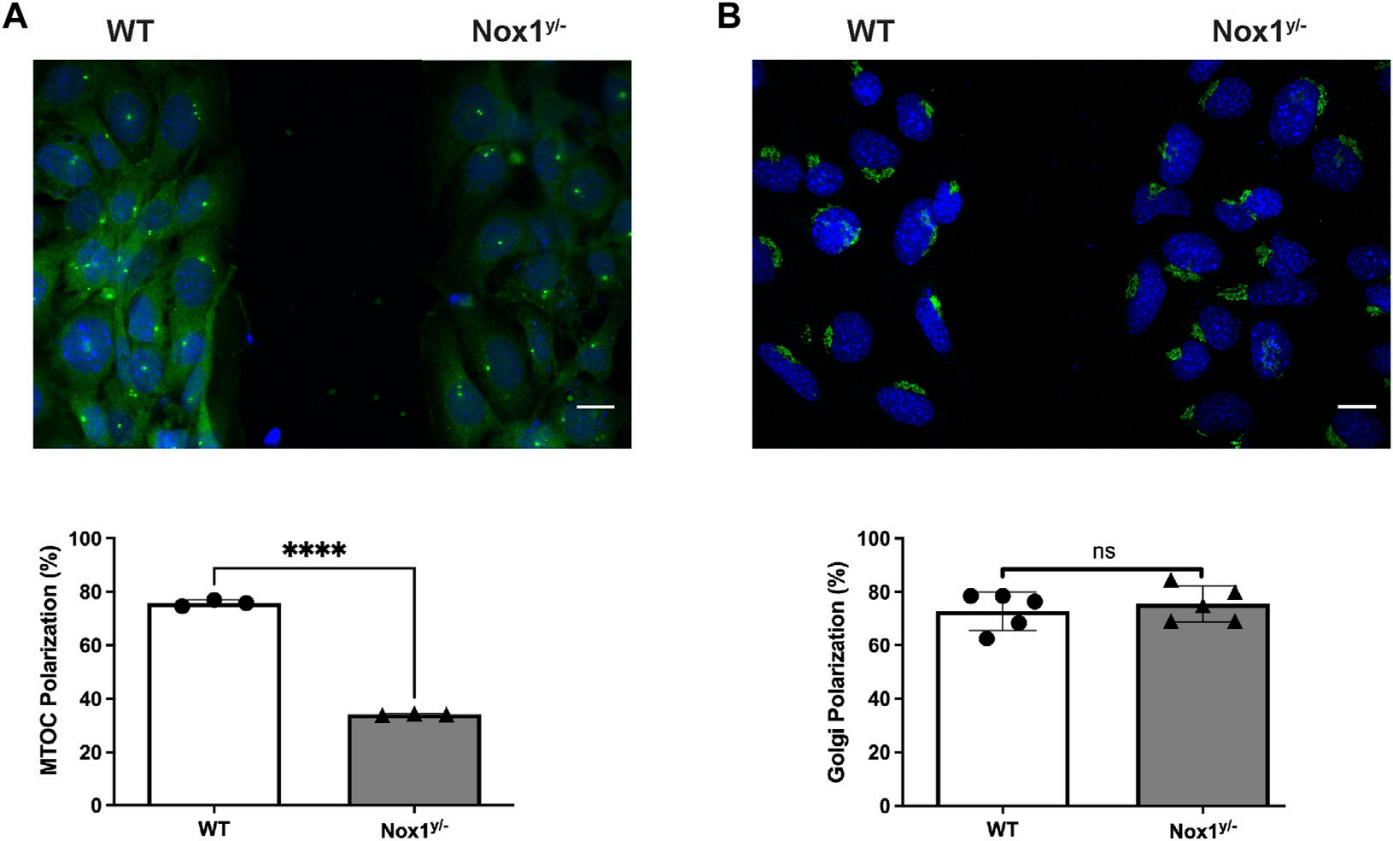
Nox1 is required for MTOC polarization. WT and Nox1^y/-^ mouse embryonic fibroblast (MEFs) cells were seeded in 2 well silicone inserts (Ibidi) on Collagen-I-coated coverslips and allowed to grow to confluency overnight. Cells were serum starved for 2 h, and after removing the insert, they were allowed to migrate to the space in the presence of 10 ng/mL PDGF-BB. After 30 min, cells were fixed and stained for **(A)** γ-Tubulin (MTOC, green), **(B)** GM130 (Golgi, green), and nucleus (DAPI, Blue). Graphs show the quantification of cells that polarize the MTOC **(A)** or Golgi **(B)** in front of the nucleus facing the wound area. Differences between genotypes were analyzed with unpaired t-test (****p < 0.0001, n = 3 for MTOC, and n = 5 for Golgi. 50–80 cells were counted in each experiment). Scale bar = 50 μm.

Previous work by our laboratory and other researchers have established that Nox1 activity is required for protrusion extension at the leading edge (San Martin et al., 2008; Maheswaranathan et al., 2011) and directional persistence (Sadok et al., 2009) during migration. Because the establishment of agonist-induced polarity precedes cell protrusion formation and is required for persistence during migration, we posited that Nox1-derived ROS are required for establishing agonist-dependent cell polarity. To examine this hypothesis, we assessed one of the first manifestations of cell polarization: the redistribution of the Golgi apparatus and the microtubule organization centers (MTOCs). Using a wound-healing assay, we found that in wild-type MEFs, PDGF induces the reorientation of the Golgi apparatus and the centrosome towards the wounded area at the front of the nucleus (Figure 1A). Interestingly, while correctly positioning their Golgi apparatus toward the wounded area, Nox1-deficient cells failed to redistribute the MTOC properly (Figure 1B).

**FIGURE 2:**
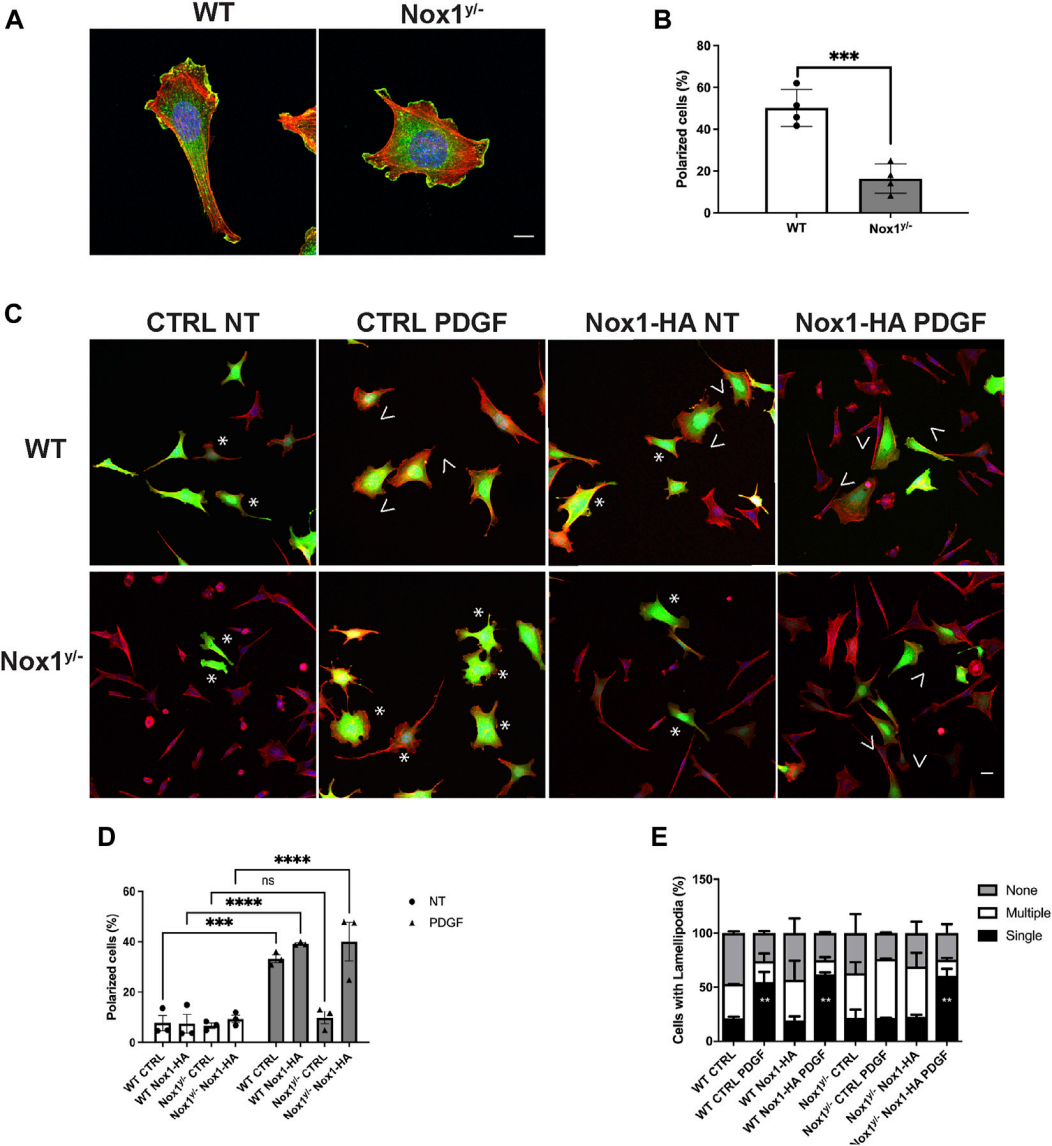
Nox1 is required for PDGF-induced polarized lamellipodia formation. WT and Nox1^y/-^ mouse embryonic fibroblasts (MEFs) were seeded on collagen-I-coated coverslips, serum starved for 16 h, and stimulated with 10 ng/mL PDGF-BB for 30 min. Cells were fixed and stained for cortactin (green), F-actin (phalloidin, red), and nucleus (DAPI, blue). **(A)** Representative images of morphology observed for WT and Nox1^y/-^ cells. Scale bar = 10 μm. **(B)** Quantification of cells that showed a polarized shape. Statistical significance was analyzed with an unpaired t-test (***p < 0.001, n = 4 and ∼150 cells analyzed per experiment). We considered the cells polarized when they showed a rear-to-front shape, including a tail and a single lamellipodium at the leading edge (as in A, WT panel). **(C)** Cells were infected with a control virus (CTRL) or a virus expressing Nox1-HA. After 24 h of infection, cells were plated and treated as described above. Staining was performed for F-actin (phalloidin, red), and infected cells showed green fluorescence. Arrowheads show cells with polar shapes (single lamellipodium), and stars show cells with multiple lamellipodia. Scale bar = 10 μm **(D)** Quantification of cells that showed a polarized shape. A two-way ANOVA test with multiple comparison was used to determine statistical significance from three independent experiments (n = 3) where ∼585 cells were analyzed in each experiment (***p < 0.001, ****p < 0.0001, ns: no significant). **(E)** Quantification of cells with single lamellipodium (black bars), multiple lamellipodia (white bars), or no lamellipodia (grey bars). Data were analyzed with a one-way ANOVA within each phenotype. Each group was compared with the control sample in basal (WT) (n = 4, ∼585 cells per experiment; **p <0.01).

**FIGURE 3:**
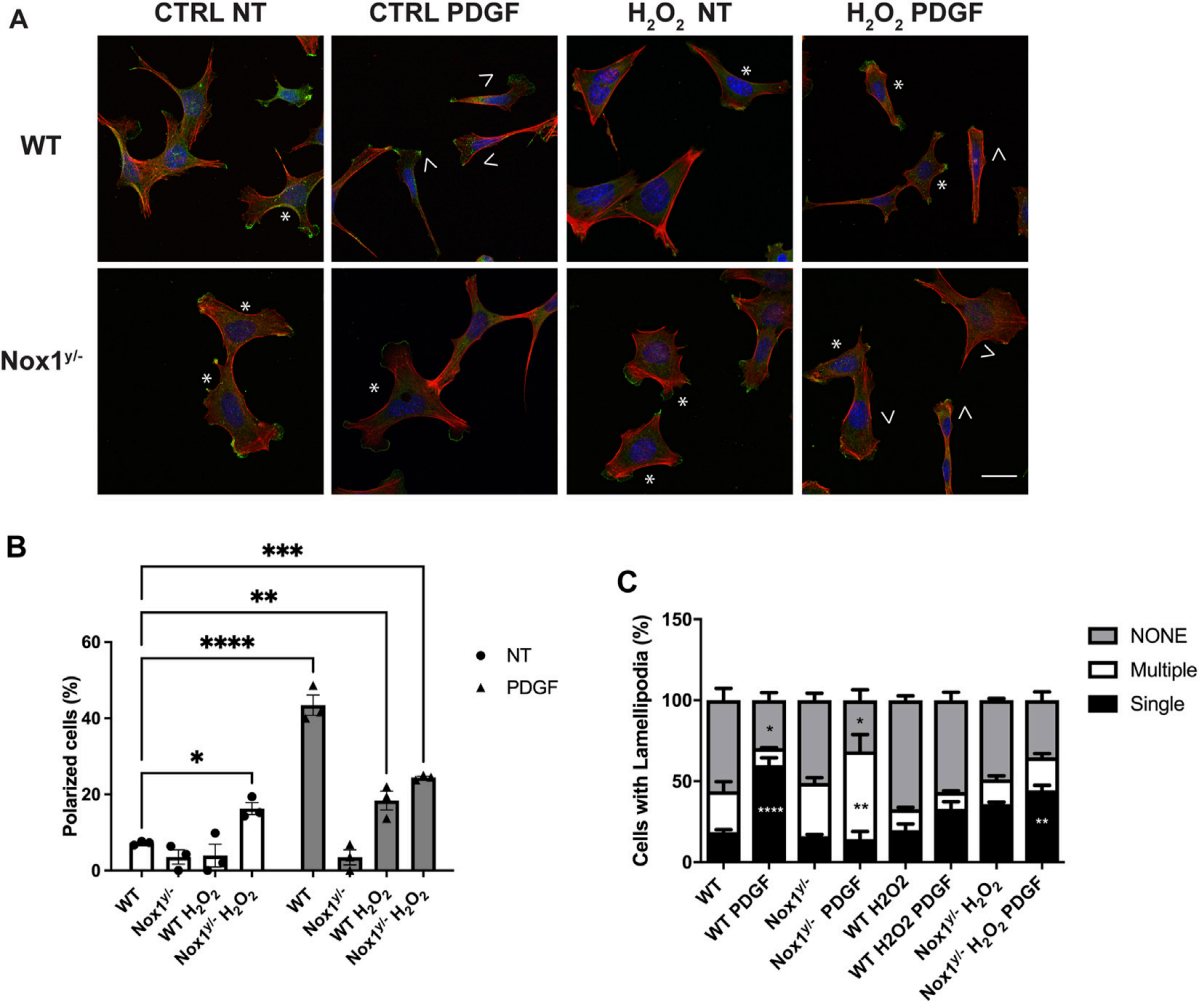
Nox1 effect on polarity and lamellipodia number is rescued with H_2_O_2_. MEF cells were seeded on collagen-I-coated coverslips, serum starved for 16 h, and stimulated with 10 ng/mL PDGF-BB in the presence or absence of 10 μm H_2_O_2_. Cells were fixed and stained for cortactin (green), F-actin (phalloidin, red), and nucleus (DAPI, blue). **(A)** Representative images of morphology observed for WT and Nox1^y/-^ cells. Arrows show cells with polar shapes (single lamellipodium), and stars show cells with multiple lamellipodia. Scale bar = 10 μm **(B)** Quantification of cells that showed a polarized shape, including a single lamellipodium. A two-way ANOVA test with multiple comparison was used to determine statistical significance from three independent experiments (n = 3) where ∼320 cells were analyzed in each experiment (*p < 0.05, **p < 0.02, ***p < 0.0002, ****p < 0.0001). **(C)** Quantification of cells with single lamellipodium (black bars), multiple lamellipodia (white bars), or no lamellipodia (grey bars). Data were analyzed with a one-way ANOVA within each phenotype. Each group was compared with the control sample in basal (WT) (n = 3, 320 cells per experiment; **p < 0.01, ***p<0.001).

It is believed that the orientation of the MTOC dictates the polarity of the microtubule (MT) network, which is critical for establishing the leading edge (Schutze et al., 1991; Palazzo et al., 2001). We observed that Nox1 deficient cells fail to relocate their MTOC properly, thus, prompting us to investigate if the formation of polarized lamellipodia was affected by Nox1 expression. We found that, in wild-type cells, 30 min of PDGF treatment induces a front-to-rear polarization characterized by a single polarized lamellipodium and a distinguishable tail (Figures 2A,B). In contrast, we observed that in Nox1 deficient cells, PDGF treatment does not produce a front-to-rear polarity axis, inducing multiple lamellipodia-like structures instead (Figures 2A,B). This aberrant phenotype was indeed due to a lack of Nox1 activity since cell polarization was recovered by re-expression of Nox1 (Figures 2C–E) or by exogenous addition of hydrogen peroxide (H_2_O_2_, 10μM, Figures 3A–C). These data demonstrate that Nox1-produced ROS is required to form a single polarized lamellipodium.

### 3.2 Nox1-deficient cells display exacerbated activation of the Par3/aPKC/Tiam complex

Several Partitioning-defective (PAR) proteins control polarization during cell migration in fibroblasts, astrocytes, and T-cell (Ludford-Menting et al., 2005; Wang et al., 2012). The Par3 homolog (Par3) polarity protein has been directly implicated in regulating centrosome re-localization during cell polarization and lamellipodium formation at the leading edge (Schmoranzer et al., 2009; Hong et al., 2010). The active Par3/aPKC complex couples with the guanine nucleotide exchange factor (GEFs) Tiam1 at the leading edge of migrating cells to activate Rac, which induces lamellipodia formation and cell migration (Etienne-Manneville and Hall, 2001; 2003).

**FIGURE 4:**
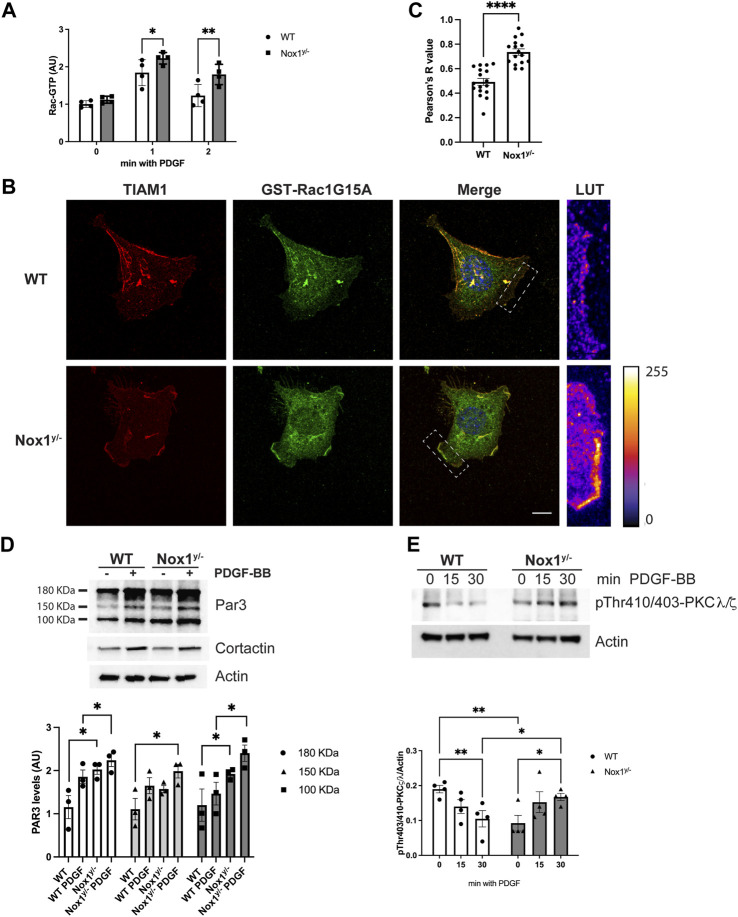
Nox1 controls Par3 activity. **(A)** Rac activity was determined using GLISA assay in WT and Nox1^y/−^cells after PDGF-BB stimulation for the indicated times. Differences between genotypes was analyzed with one-way ANOVA (*p < 0.05, **p < 0.01, n = 4). **(B)** WT and Nox1^y/-^ MEFs, were seeded on collagen-I-coated coverslips, serum starved for 16 h, and stimulated with 10 ng/mL PDGF-BB for 30 min. Cells were fixed and incubated with GST-Rac1G15A and then immunostained for GST and Tiam. Tiam activation depended on Nox1 and was tracked by colocalization of GST, and Tiam signals at the membrane of lamellipodia and protrusions. Look up table (LUT) panels correspond to the magnification of the lamellipodia area shown on merge images (white boxes). Scale bar = 10 μm **(C)** Quantification of colocalization between Tiam and GST-Rac1G15A signal. The graph shows Pearson’s R values at the lamellipodium area for WT and protrusion area for Nox1^y/-^ cells. Differences were evaluated with an unpaired t-test (****p < 0.001, n = 3, and 5-7 cells per condition in each independent experiment). **(D)** MEF cells were serum starved for 16 h and stimulated with 10 ng/mL of PDGF-BB for 3 min. Phosphorylated proteins were isolated by affinity chromatography and analyzed by immunoblot using an antibody against Par3. Cortactin and actin were used as controls. Graph shows densitometric analysis of observed levels of phosphoproteins for the 180, 150, and 100 KDa bands corresponding to different Par3 isoforms from three independent experiments. Statistical significance was evaluated with a two-way ANOVA (*p < 0.05, n = 3). **(E)** Cells were serum starved for 16 h and stimulated for 15 and 30 min with 10 ng/mL PDGF-BB. Cell lysates were analyzed for immunoblot for pThr410/403-PKCζ/λ corresponding to the activation loop of atypical PKCs. Graphs show the densitometric analysis of four independent experiments. Statistical significance was evaluated with a two-way ANOVA (*p < 0.02, p**<0.003, n = 4).

Since Nox1-deficient cells display altered lamellipodia and centrosome polarization, we posited that Nox1 is implicated in the Par3/aPKC/Tiam/Rac complex activation. We first quantified Rac activity before and after PDGF treatment in WT and Nox1^y/-^ cells to test this hypothesis. We found that in basal the levels of active Rac (Rac-GTP) are similar in both genotypes. However, after 1 and 2 min with PDGF, Nox1^y/-^ cells showed significantly higher levels of Rac-GTP compared with WT cells (Figure 4A). The differential Rac activation observed between WT and Nox1y/- cells lead us to look for active Tiam in the lamellipodia. We visualized the localization of active Tiam1 through its colocalization with the Rac nucleotide-free mutant GST-Rac1G15A, known to bind with high affinity to the active GEFs (Valdivia et al., 2020). We observed the highest levels of colocalization between Tiam1 and GST-Rac1G15A in the multiple lamellipodia-like protrusions of Nox1^y/-^ cells, while in WT cells, the colocalization was limited to the main lamellipodium (Figures 4B,C). We used recombinant GST protein (Supplementary Figure S3) and a non-immune IgG (data not shown) as specificity controls.

Numerous studies have shown that Par3 binding capacity is regulated by phosphorylation. Par3 phosphorylation can block or facilitate the recruitment of binding partners. Par3’s binding to Tiam and the ability to localize to the cells’ edge are associated with its phosphorylation status (Hurd et al., 2003; Goldstein and Macara, 2007; Ling et al., 2010). Therefore, we evaluated Par3 phosphorylation by selectively purifying phosphorylated proteins from wild-type and Nox1^y/-^ cells before and after PDGF stimulation. The isolated phosphoproteins and phosphoprotein-containing complexes were analyzed by immunoblots using specific antibodies. In wild-type cells, PDGF induces the phosphorylation of the two spliced variants of Par3 (180kDa and 100 kDa), while in Nox1 deficient cells, we observed exacerbated phosphorylation of Par3 after PDGF stimulation (Figure 4D). In contrast, PDGF-induced phosphorylation of cortactin reached a similar level in both genotypes (Figure 4D), indicating that the increase in Par3 phosphorylation is unlikely to result from a widespread increase in protein phosphorylation.

Par3 is phosphorylated by the aPKC (Lin et al., 2000; Li et al., 2010; Morais-de-Sa et al., 2010). Thus, we investigated whether Nox1 expression affects aPKC activity. We found the phosphorylation of the activation loop of the aPKC (PKCζ/λ) decreased after PDGF stimulation in WT whereas it increased in Nox1^y/-^ cells (Figure 4E).

Together, these data indicate that Nox1^y/-^ cells display a higher activation of the Par3/aPKC complex, probably driven by hyperphosphorylation.

### 3.3 PP2A is inactivated by Nox1 deficiency

Several protein phosphatases have been associated with the de-phosphorylation of the Par3/aPKC complex (Nam et al., 2007; Traweger et al., 2008; Krahn et al., 2009; Ogawa et al., 2009; Schumann et al., 2012). In particular, it is well established that the aPKC is a substrate for the protein phosphatase 2A (PP2A) (Nunbhakdi-Craig et al., 2002). Since aPKC shows increased phosphorylation in Nox1^y/-^ cells, we tested the hypothesis that PP2A undergoes inactivation in Nox1-deficient cells.

**FIGURE 5:**
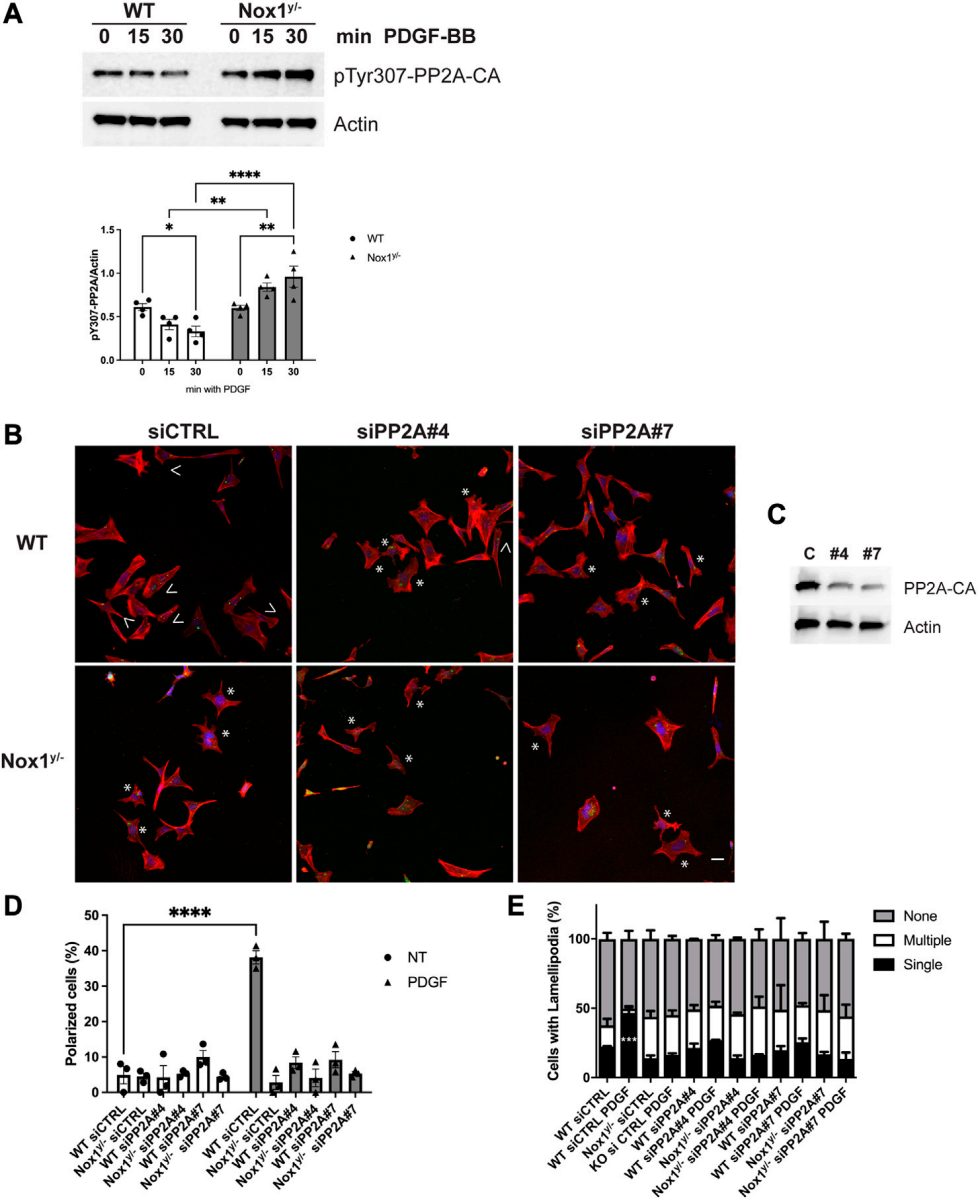
Nox1 regulates polarity through PP2A phosphatase activity. **(A)** Cells were serum starved for 16 h and stimulated for 15 and 30 min with 10 ng/mL PDGF-BB. Cell lysates were analyzed for immunoblot for the **i**nactivating-phosphorylation of PP2A-CA using an antibody against pTyr307. Graphs show the densitometric analysis of 4 independent experiments. Data were analyzed with a two-way ANOVA (*p < 0.05, **p < 0.01, ****p < 0.0001, n = 4). **(B)** Cells were co-transfected with siGlo and siRNA Control (CTRL) or siRNA against PP2A-CA (sequence #4 or #7). Representative images of morphology observed for WT and Nox1^y/-^ cells. Arrows show cells with polar shapes (single lamellipodium), and stars show cells with multiple lamellipodia. Scale bar = 10 μm **(C)**. Efficiency of knockdown using the siRNA Control (CTRL) or siRNA against Par3 (sequence #4 or #7). **(D)** Quantification of cells that showed a polarized shape, including a single lamellipodium. Statistical differences were determined with two-way ANOVA from three independent experiments (n = 3). Each group was compared (n = 3, 200 cells, ****p < 0.001). **(E)** Quantification of cells with single lamellipodium (black bars), multiple lamellipodia (white bars), or no lamellipodia (grey bars). Data were analyzed with a one-way ANOVA within each phenotype. Each group was compared with the control sample in basal (WT) (n = 3, 200 cells; ***p < 0.001).

Several post-translational modifications regulate PP2A: one is the Tyr 307 phosphorylation within the catalytic subunit of PP2A (PP2A-CA), which is associated with loss of phosphatase activity (Chen et al., 1992). Figure 5A shows that PP2A-CA Tyr 307 phosphorylation decreases shortly after PDGF stimulation in WT while increasing in Nox1^y/-^ cells. This result indicates that after PDGF stimulation, PP2A is activated in the wild type while it is inhibited in Nox1^y/-^ cells. Furthermore, the pattern of PP2A inactivation mirrors the phosphorylation in the activation loop of the aPKC (Figure 4E), suggesting that increased aPKC phosphorylation in Nox1-deficient cells is the result of PP2A inactivation.

**FIGURE 6:**
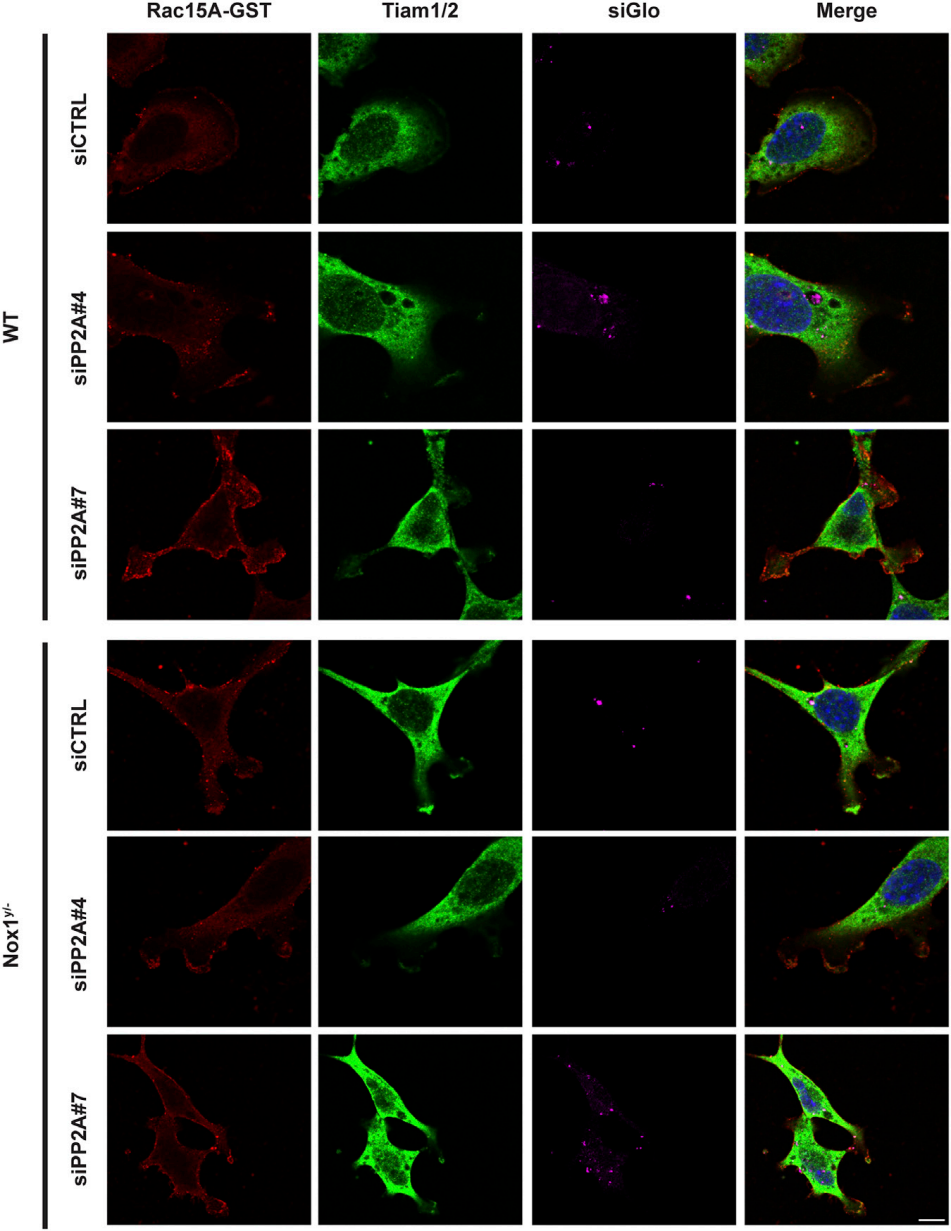
PP2A expression affects GST-Rac1G15A/Tiam colocalization at the lamellipodium. WT and Nox1^y/-^ MEFs, were seeded on collagen-I-coated coverslips, co-transfected with siGlo and siRNA against PP2A CA (sequence #4 or #7: siPP2A#4 and siPP2A#7) or siRNA control (siCTRL), serum starved for 16 h and stimulated with 10 ng/mL PDGF-BB for 30 min. Cells were fixed, incubated with GST-Rac1G15A, and then stained for GST and Tiam. Representative images are shown. Tiam activation at the lamellipodia area is observed in yellow by the colocalization of GST and Tiam. Scale bar = 10 μm .

**FIGURE 7:**
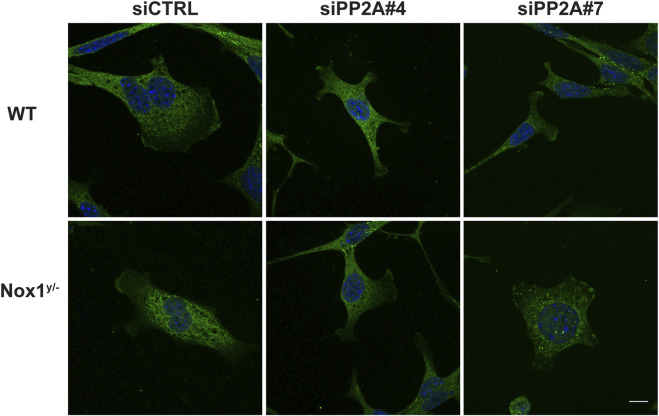
PP2A controls Par3 localization. WT and Nox1^y/-^ cells were co-transfected with siGlo and siRNA Control (CTRL) or siRNA against PP2A-CA (sequence #4 or #7: siPP2A#4 and siPP2A#7), serum starved for 16 h and stimulated with 10 ng/mL PDGF-BB for 30 min. Cells were fixed and immunostained for Par3 (green) and nucleus (DAPI, blue). Representative images of Par3 localization observed for WT and Nox1^y/-^ cells. Scale bar = 10 μm.

Consistent with a role for PP2A phosphatase activity in agonist-mediated polarization downstream of Nox1, wild-type cells treated with siRNA against PP2A-CA exhibit the same phenotype of aberrant polarization (Figures 5B–E) and increased Tiam localization to lamellipodia-like protrusions (Figure 6) as Nox1-deficient cells. Regarding the localization of the polarity complex, we observed that, in control cells, Par3 is distributed to the edge of the lamellipodium while amassing at the edge of multiple small lamellipodia-like structures in Nox1^y/-^ cells and in wild-type cells when PP2A-CA expression is silenced (Figure 7).

**FIGURE 8:**
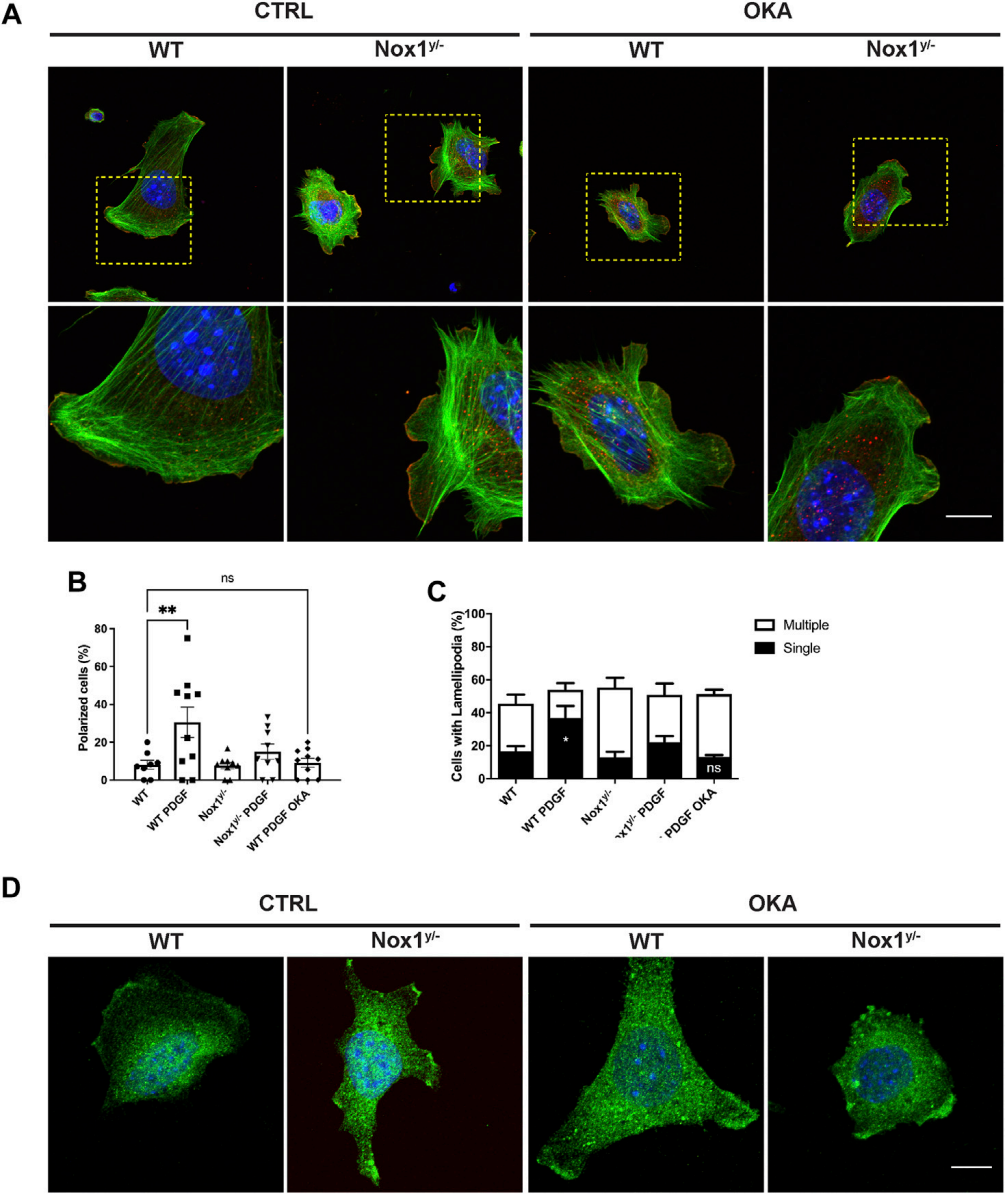
Okadaic acid affects cell polarity, the number of lamellipodia, and Par3 localization in lamellipodia. Cells were seeded on Collagen I-coated coverslips, allowed to attach, and serum starved for 16 h. Then, they were incubated with 1 μM of Okadaic acid (OKA) for 30 min and stimulated with 10 ng/mL of PDGF-BB for an additional 30 min. Cells were fixed and stained for **(A)** Cortactin (red), F-actin (phalloidin, green), and nucleus (DAPI, blue). The lower panel corresponds to the magnification of the above pictures, showing F-actin distribution in single lamellipodia and protrusion in WT and NOX1^y/-^ cells with and without OKA. Scale bar = 10 μm. **(B)** Quantification of the percentage of polarized cells and **(C)** Number of lamellipodia after OKA treatment. Percentage of polarized cells **(B)** and number of lamellipodia **(C)** were calculated from images of 8–10 random field of view per condition (807 cells total from three independent experiments). Data were analyzed with one-way ANOVA (*p < 0.05, **p < 0.01, ns: no significant). **(D)** Cells were treated as before and stained with an antibody raised against Par3 (green) and DAPI for nucleus (blue). Representative pictures show the distribution of Par3 along the lamellipodia of WT and Nox1^y/-^ cells in basal and OKA-treated cells. Scale bar = 10 μm.

Finally, we observed that in the presence of the PP2A inhibitor okadaic acid (OKA, 1 μM), PDGF treatment in wild-type cells accurately recapitulates the phenotype of aberrant polarization (Figures 8A–C) and Par3 accumulation observed in Nox1 deficient cells (Figure 8D). Because OKA has no effect in Nox1 deficient cells, this experiment suggests that protein phosphatase activity is already inhibited and cannot be inhibited further in Nox1^y/-^ cells. This result is consistent with the idea that PP2A is a downstream effector of Nox1-induced cell polarization after PDGF treatment.

**FIGURE 9:**
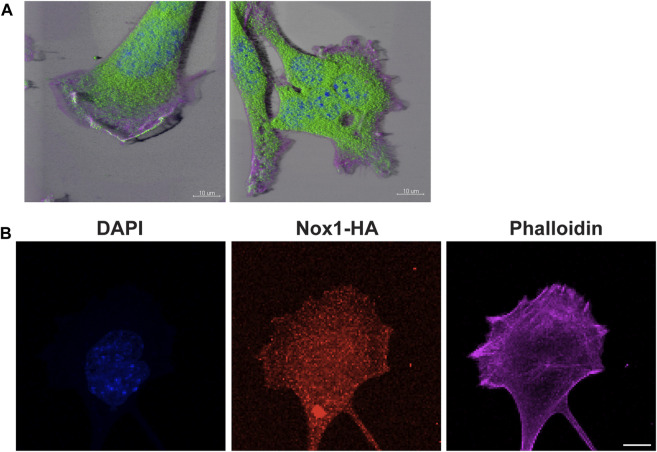
PP2A and Nox1-HA localize in lamellipodium. **(A)** WT and Nox1^y/-^ cells seeded and treated as described before, were stained for PP2A-CA (green), nucleus (blue), and F-actin (magenta). 3D images were created using Imaris software. Bar = 10 μm. **(B)** WT cells were infected with Nox-1-HA adenovirus. After 24 h, Nox1 localization in cells was assessed by using an anti-HA antibody (red) and phalloidin (magenta), Scale bar = 10 μm.

To further confirm the role of PP2A activity downstream of Nox1 in lamellipodia formation, we observed that endogenous PP2A-CA localized at the edge of lamellipodium in WT cells while getting excluded from the multiple lamellipodia in Nox1^y/-^ cells (Figure 9A). Although H_2_O_2_ can quickly diffuse in the cell, our results imply a proximity-based relationship between Nox1 and PP2A. Since Nox1 is expressed in low amounts in MEFs, making it hard to detect, we overexpressed Nox1 with an HA-tag. By staining for the HA tag, we observed that Nox1-HA localizes to the lamellipodia and to lateral zones at the edge of the cell (Figure 9B). Additionally, to rule out the possibility that reactive oxygen species (ROS) derived from Nox1 could affect the activity of PP2A, we used the thiol-reactive probe 5-IAF (5-iodoacetoamidofluorescein). Nox1 expression levels did not affect the redox status of PP2A-CA (Supplementary Figure S4).

These experiments demonstrate that PP2A is not localized at the proximity of the leading edge and that its activity is inhibited in Nox1-deficient cells. Furthermore, PP2A activity is responsible for the agonist-induced aberrant polarity in these cells.

### 3.4 Nox1 effects are also observed in primary mouse aortic smooth muscle cells (MASMCs) and femoral artery neointimal hyperplasia

After vascular injury, PDGFR beta activation by PDGF induces vascular smooth muscle migration and contributes to vascular diseases (Banai et al., 1998; Kohno et al., 2013; Guan et al., 2014; Wu et al., 2021). In addition, PDGF also increases ROS production by activating Nox1 (Marumo et al., 1997; Lassegue et al., 2001; Lavigne et al., 2001). Because Nox1 expression increases after injury and is required for the injury-induced formation of neointima in small animal models (Lee et al., 2009; Xu et al., 2012), we speculate that our signaling pathway is conserved in this cell type.

**FIGURE 10:**
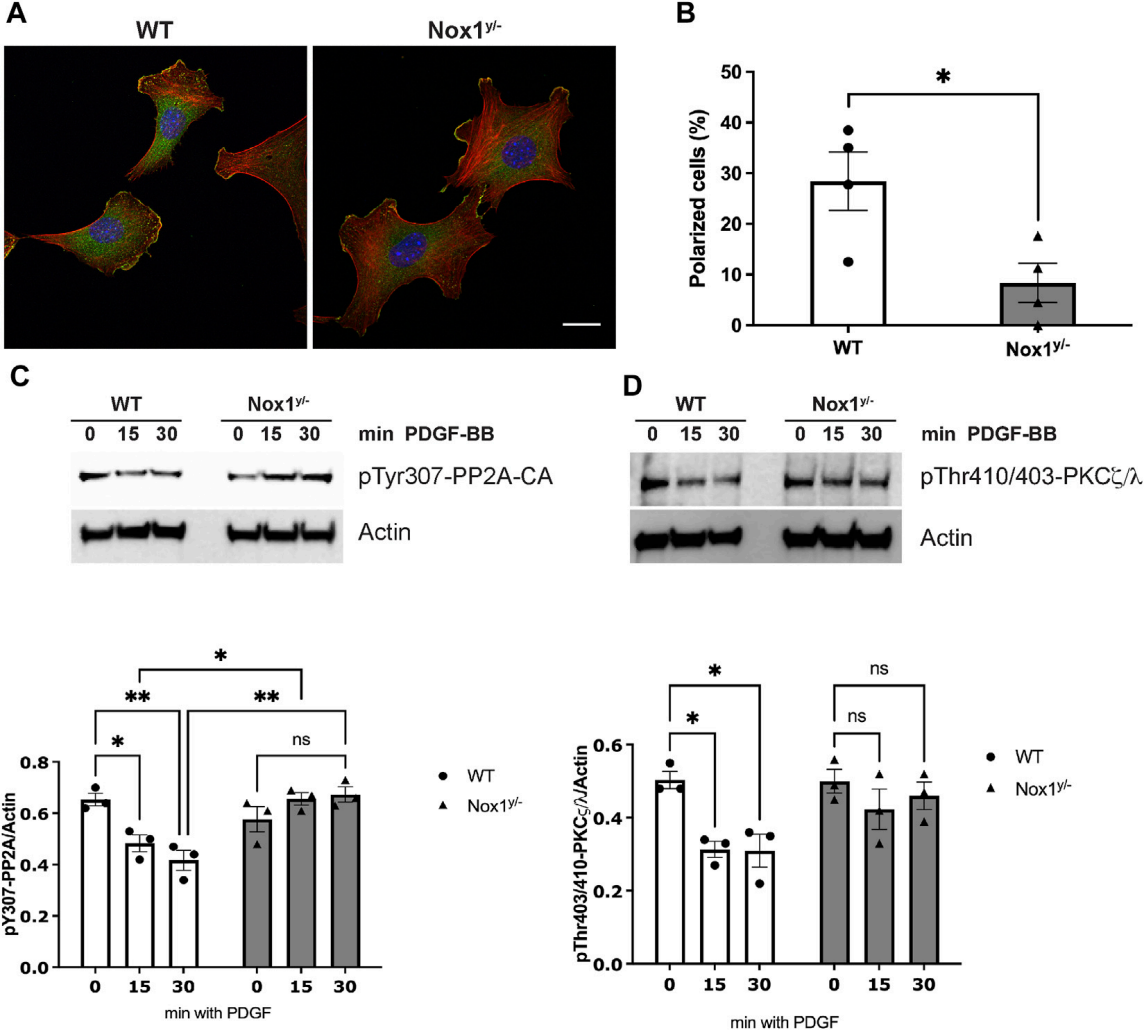
Nox1 effect on polarity is observed in vascular smooth muscle cells. **(A)** Primary mouse aortic smooth muscle cells (MASMCs) derived from WT and Nox1^y/-^ mice were seeded on collagen-I-coated coverslips, serum starved for 16h, and stimulated with 10 ng/mL PDGF-BB for 30 min. Cells were fixed and stained for cortactin (green), F-actin (red phalloidin), and nucleus (DAPI, blue). Scale bar = 10 μm **(B)** graph shows the quantification of cells that showed a polarized shape, including a single lamellipodium. Statistical significance was analyzed with an unpaired t-test (*p < 0.005, n = 4 and ∼50 cells analyzed per experiment). MASMCs cells were serum starved for 16 h and stimulated for 15 and 30 min with 10 ng/mL PDGF-BB. Cells lysates were analyzed by immunoblot for the **(C)** inactivating-phosphorylation of PP2A-CA using an antibody against pTyr307 and for **(D)**. pThr410/403-PKCζ/λ corresponding to the activation loop of atypical PKCs. Graphs below each figure show the densitometric analysis of 4 independent experiments (*p < 0.05, **p < 0.01).

Indeed, as we observed in mesenchymal cells (MEFs), Nox1 depletion affected the polarization of primary mouse aortic smooth muscle cells (MASMCs) in response to PDGF, also inducing multiple lamellipodia in these cells (Figures 10A,B). Accordingly, Nox1^y/-^ MASMC also showed an aberrant inactivation of PP2A after PDGF treatment, consistent with exacerbated atypical PKCζ/λ activation (Figures 10C,D).

**FIGURE 11:**
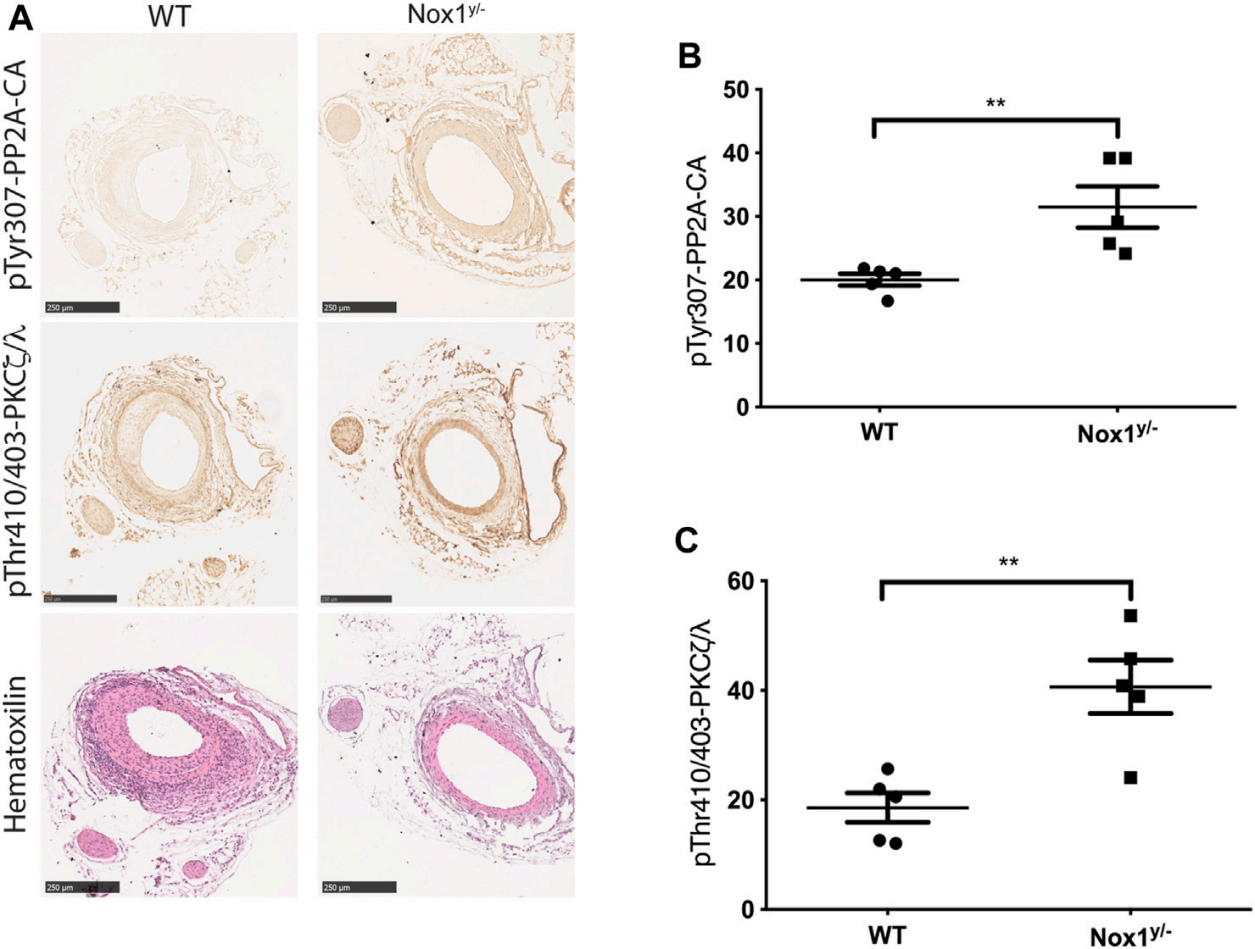
Nox1 effect on the inactivation of PP2A phosphatase and activation of PKCζ/λ is also observed in the femoral arteries of wire-injured mice. Femoral arteries from WT and Nox1^y/-^ mice were injured using a wire, as described in the Material and Methods section. After 21 days, arteries were carefully excised and processed for histological analysis. **(A)** Sequential sections from 5 animals were used for Hematoxylin and Eosin staining and for the inactivating-phosphorylation of PP2A-CA using an antibody against pTyr307 and for pThr410/403-PKCζ/λ corresponding to the activation loop of atypical PKCs. Graphs show the relative intensity of DAB staining assessed using ImageJ. For pTyr307-PP2A-CA **(B)** and pThr410/403-PKCζ/λ **(C)**. Differences were assessed with a t-test (n = 5, **p < 0.001), Scale bar = 250 μm.

It is well-accepted that vascular smooth muscle cell migration contributes to the injury-induced neointimal formation (Inoue and Node, 2009). Therefore, to evaluate the physiological relevance of our pathway *in vivo*, we performed the murine model of femoral artery wire injury followed by histological analysis. Our results showed that the inhibition of neointimal hyperplasia in Nox1^y/-^ mice was accompanied by increased staining for the inactive form of PP2A-CA and an increased signal for active aPKCζ/λ (Figures 11A–C). These results suggest that the inactivation of PP2A and the aberrant activation of aPKC may contribute to reducing neointima formation observed in the Nox1^y/-^ mice arteries.

## 4 Discussions

Cell migration is essential for many biological processes, including embryological development, tissue architecture, immune surveillance, angiogenesis, and wound healing (Escandon et al., 2022). Similarly, cell migration contributes to pathological processes such as atherosclerosis and restenosis (Schwartz, 1997). Establishing cell polarity with a well-defined trailing edge and forming a single lamellipodium at the cell’s leading edge is essential for directed cell migration and is a hallmark of mesenchymal cell migration.

Previous studies by us and others have shown that Nox1 deficiency inhibits cell migration in various cell types, including fibroblast and smooth muscle cells. Here we further characterize the mechanistic role of Nox1 during migration and show that Nox1^y/-^ cells treated with PDGF fail to properly reorient their MTOC toward the front to initiate directional migration. Surprisingly, these cells still correctly position the Golgi apparatus and function by distributing cargo to the plasma membrane since we do not observe differences in the amount of PDGF-R at the membrane. The Golgi apparatus can nucleate and stabilize non-centrosomal microtubules to regulate their position, shape, and polarized cargo transport (Zhu and Kaverina, 2013; Rios, 2014; Sanders and Kaverina, 2015). However, Golgi-derived microtubule arrays are nucleated, stabilized, and tethered differently than the microtubules in the MTOC. Therefore, our observation suggests that the Nox-1 effect may be directed to MTOC rather than Golgi microtubules.

We also showed that Nox-1-derived ROS is necessary for a single lamellipodium formation and that the lack of Nox1 leads to multiple lamellipodium-like structures. Previous work has posited Nox1-derived ROS as responsible for actin polymerization at the lamellipodia [reviewed in (San Martin and Griendling, 2010)]. In this case, the initial ROS burst produced by Nox1 after PDGF stimulation activates Src > PAK > LIMK pathway to induce cofilin inactivation (F-actin stabilization). Here we described a new function for Nox1-produced ROS dictating the formation of a single polarized lamellipodium. Although ROS can quickly diffuse through the cell, forming a single lamellipodium implies a spatial-temporal tethering of Nox1 to a particular membrane area. Accordingly, we showed that Nox1-HA is widely distributed in the cell and localizes at the lamellipodium edge. We speculate that the Par polarity complex is also responsible for recruiting Nox1 to the membrane, similar to what has been described in endothelial cells under shear stress, where Tiam acts as an adaptor between VE-cadherin, p67phox (a Nox1 and Nox2 subunit), and Par3 (Liu et al., 2013). In this case, the authors also described the local activation of Rac and ROS production (Liu et al., 2013). Many examples in the literature exist in which ROS can induce more ROS production. Since Rac activity can induce Nox1-derived ROS, it is possible that this is also the case in our system, and there is more than one ROS wave. Perhaps an initial ROS burst after PDGF stimulation is responsible for the spatial localization of the Par polarity complexes to initiate the lamellipodium formation, while a second burst is responsible for the actin polymerization driven by Nox1-SSH1L at the lamellipodium. Further experiments are necessary to rule out this possibility.

Furthermore, we also believe that the multiple lamellipodia observed after the absence of Nox1 indicate a lack of cell polarity rather than a transition from directional cell migration to amoeboid migration (Petrie et al., 2009; SenGupta et al., 2021). This is supported by the observation that in our chemotaxis assay, Nox1^-/y^ cell still showed a decreased migration rate.

Redox signaling regulates protein phosphatases activity in a positive (Kim et al., 2009; Maheswaranathan et al., 2011) and negative manner (Meng et al., 2002; Ostman et al., 2011). Our results showed that Nox1-derived ROS does not oxidizes PP2A CA, suggesting an indirect mechanism is controlling PP2A activity. Our previous work has shown that Nox1-derived ROS are required for SSH1L/14-3-3 complex disruption (Maheswaranathan et al., 2011) and activation of the phosphatase SSH1L leading to cofilin dephosphorylation (San Martin et al., 2008; Lee et al., 2009). In the present study, we demonstrate that Nox1-derived ROS is required to locate and activate the phosphatase PP2A properly. Additional research is necessary to determine if the oxidation of chaperone regulatory proteins such a 14-3-3 can also control PP2A activity.

Alternatively, redox signaling can also regulate kinase activity (Maheswaranathan et al., 2011; Rochaix, 2013; Truong and Carroll, 2013). Of relevance for our work, Src has been described as one of the kinases that phosphorylates PP2A CA Y307 (Barisic et al., 2010), and Src activity can be inhibited by ROS (Tang et al., 2005). Further experiments are necessary to corroborate that Nox1-derived ROS controls PP2A activity by inhibiting Src function.

**FIGURE 12:**
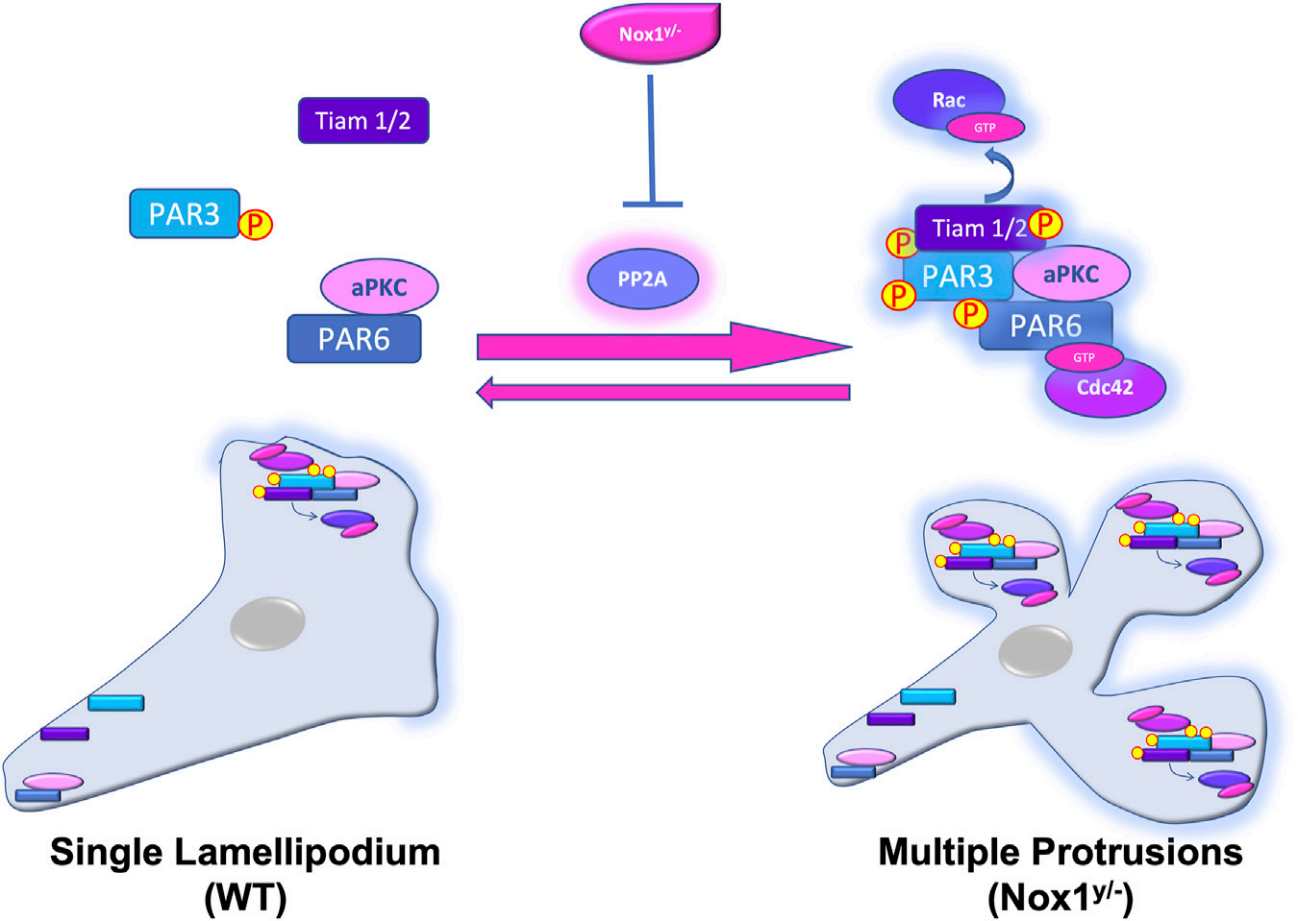
Nox1 regulates the activity and localization of the Par3/aPKC/Tiam polarity complex by regulating PP2A activity. In WT cells, PDGF stimulation induces the polarized formation of a single lamellipodium via the assembly of Par3/aPKC/Tiam and the activation of Rac at the leading edge. At the rear end of the cell, Par6 interacts with aPKC and Par3 is not binding Tiam. Our current working model propose that the lack of Nox1 (Nox1^y/-^) inhibits the activity of the phosphatase PP2A, inducing the aberrant phosphorylation and activation of the Par3/aPKC/Tiam polarity complex. Par3, PP2A, Nox1-HA, and active Tiam amass at multiple membrane locations forming multiple lamellipodia-like structures probably by Rac activation.

Our current working model propose that the lack of Nox1 inhibits the activity of the phosphatase PP2A, leading to the hyperphosphorylation of the polarity complex Par3/aPKC/Tiam. This aberrant activation, amasss the polarity complexes at the membrane leading to the formation of multiple lamellipodia-like structures (Figure 12).

In conclusion, our results here point to a specific function for Nox1 during the establishment of cell polarity driven by the Par polarity complex. Furthermore, our data support a mechanistic model in which Nox-1-induced ROS can control the phosphorylation status of aPKC and Par3 by regulating the activity of PP2A, leading to unique and functional lamellipodia.

## Data Availability

The raw data supporting the conclusion of this article will be made available by the authors, without undue reservation.
