# Supplementary material for: Nox1-based NADPH oxidase regulates the Par protein complex activity to control cell polarization
Source: Front Cell Dev Biol. 2023 Aug 11;11:1231489. doi: 10.3389/fcell.2023.1231489 (PMC10457011; doi:10.3389/fcell.2023.1231489)
Supplement: Supplementary file 1 [file DataSheet1.docx]

Supplementary Material

**
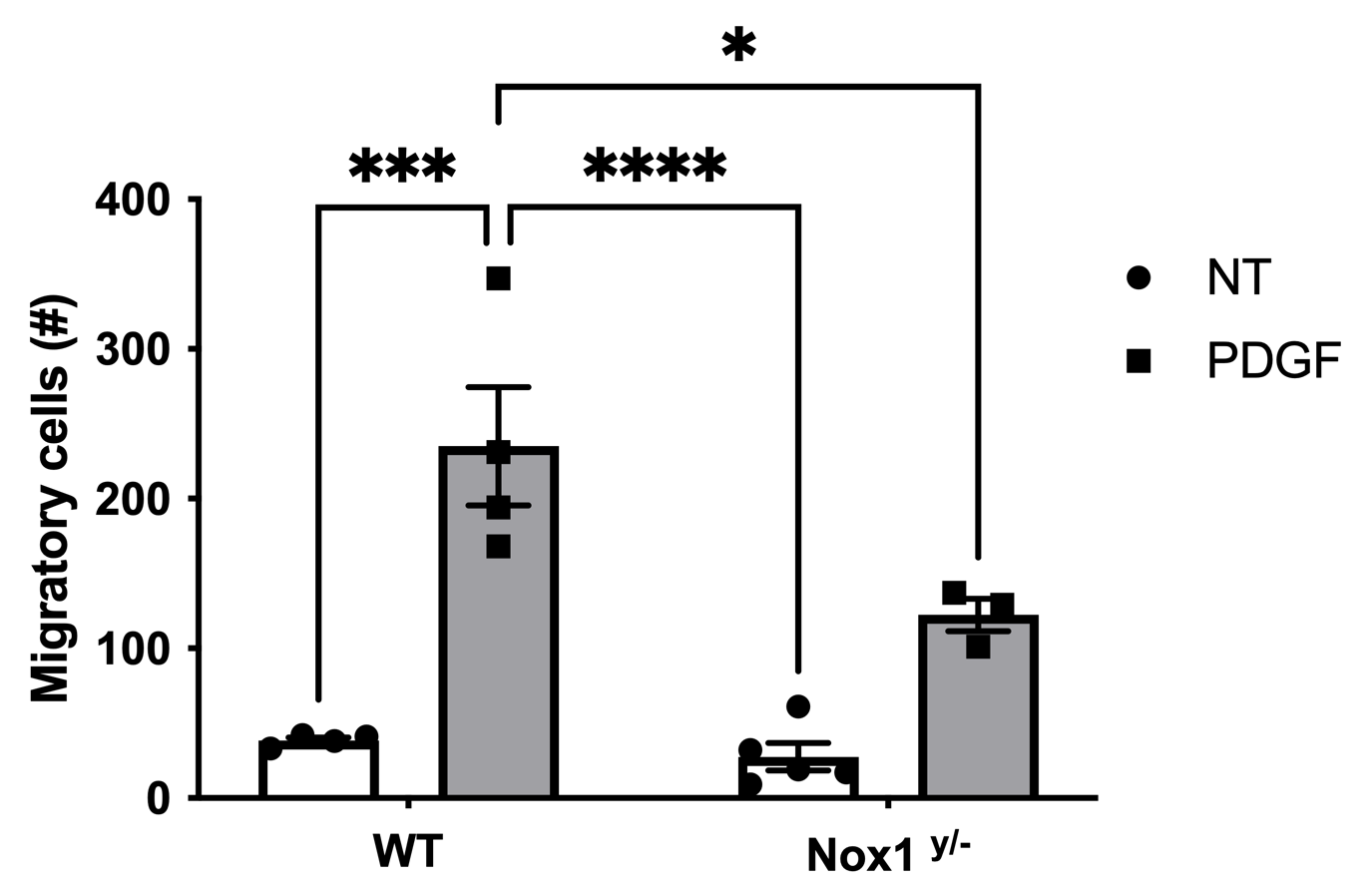
**

**Supplementary Figure 1. Nox1 deficient MEFs showed impaired PDGF-BB-induced migration.** WT and Nox1^y/-^ MEFs were seeded in a Boyden Chamber and allowed to migrate towards starvation media (NT) or 10 ng/ml PDGF-BB (PDGF) for 3 h. Graph shows the number of cells that migrated towards the filter ± S.E.M. Data was analyzed with a two-way ANOVA with Tukey test for multiple comparisons. (*p<0.05, ***p<0.001, ***p<0.0001, n=4-5).


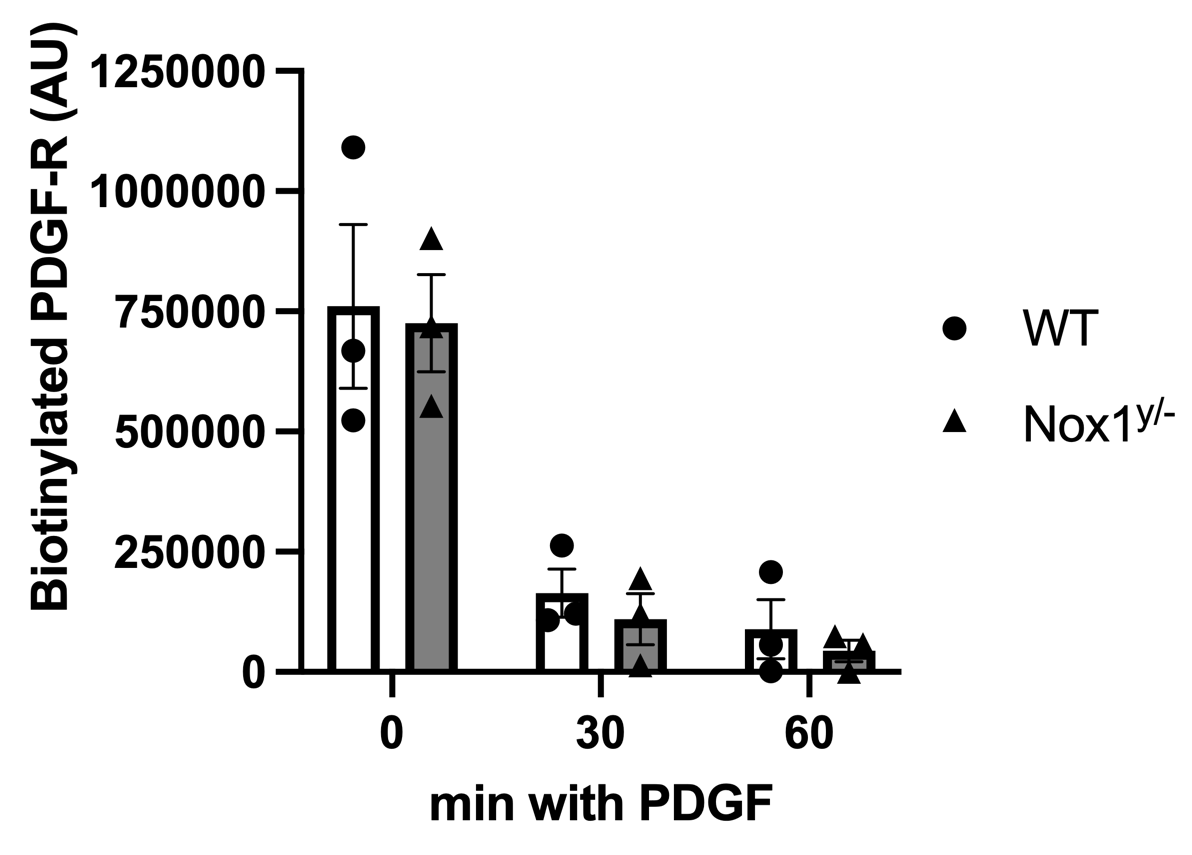


**Supplementary Figure 2. Nox1 does not affect the amount of PDGF Receptor at the membrane.** WT and Nox1^y/-^ MEFs were seeded on collagen-I-coated plates, serum starved for 16 h and stimulated with 10 ng/ml PDGF-BB (PDGF) for the indicated times. Surface proteins were biotinylated and subsequently precipitated using Avidin-beads. Precipitated proteins and total lysates were analyzed by Western blot using PDGF Receptor (PDGF-R) antibody. The graph shows the densitometric analysis of biotinylated PDGF-R standardized by its total levels of expression for 3 independent experiments (n=3). Data was analyzed with two-way ANOVA. No significant differences were observed.


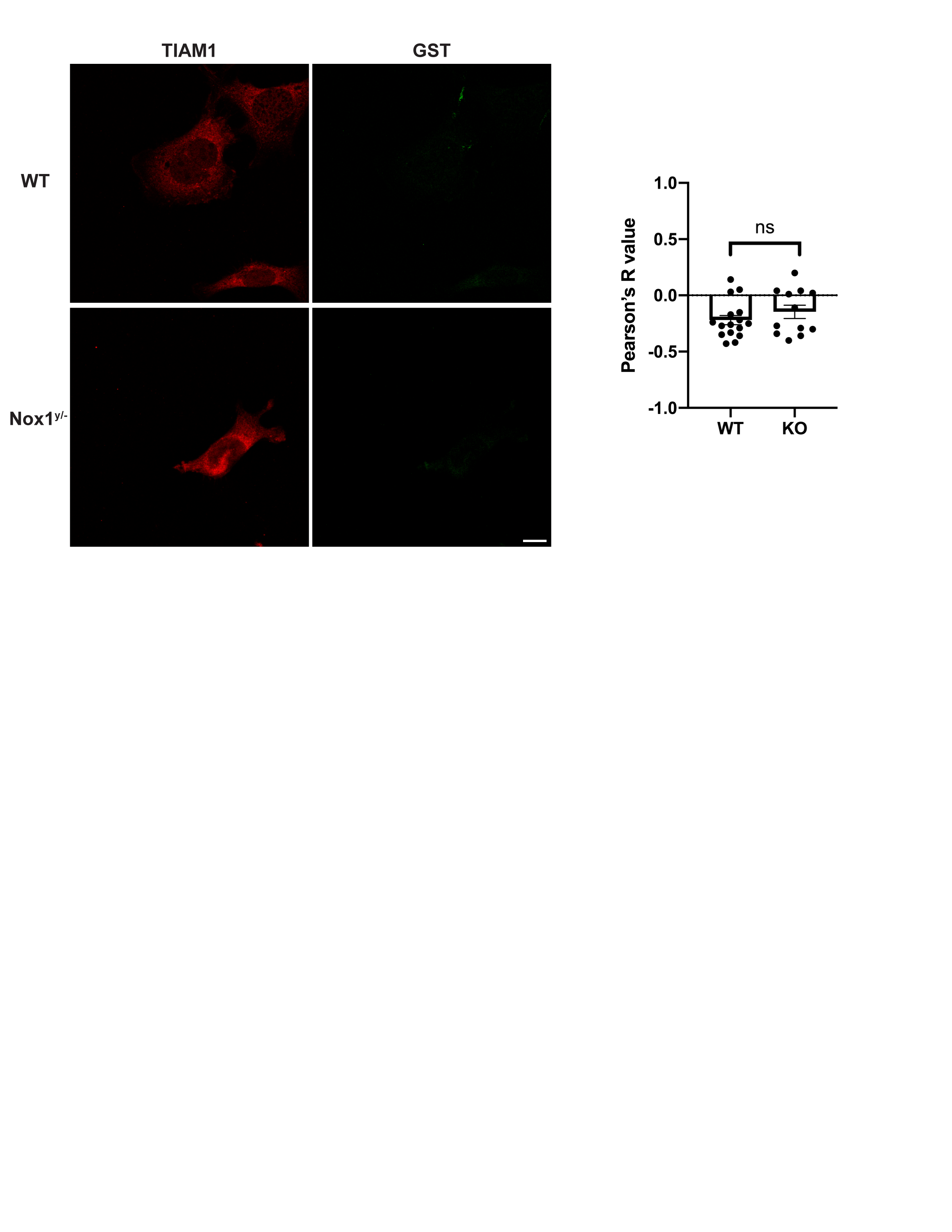


**Supplementary Figure 3. Control of specificity in far-immunofluorescence assays.** WT and Nox1^y/-^ (KO) MEFs were processed for far immunofluorescence as described in Material and Methods. In brief, samples were incubated with purified recombinant GST (negative control) and subsequently stained for Tiam (Red) and GST (green). Magnification bar = 10μm. Graph shows the colocalization Pearson’s R coefficients, calculated from specific ROI located at the membrane of lamellipodium and lamellipodia-like protrusions based on the Tiam signal. Results show the absence of GST signal (green) and colocalization between Tiam-GST in far-immunofluorescence assays (ns= non-significant, R values between 0.7-1 are considered true colocalization).


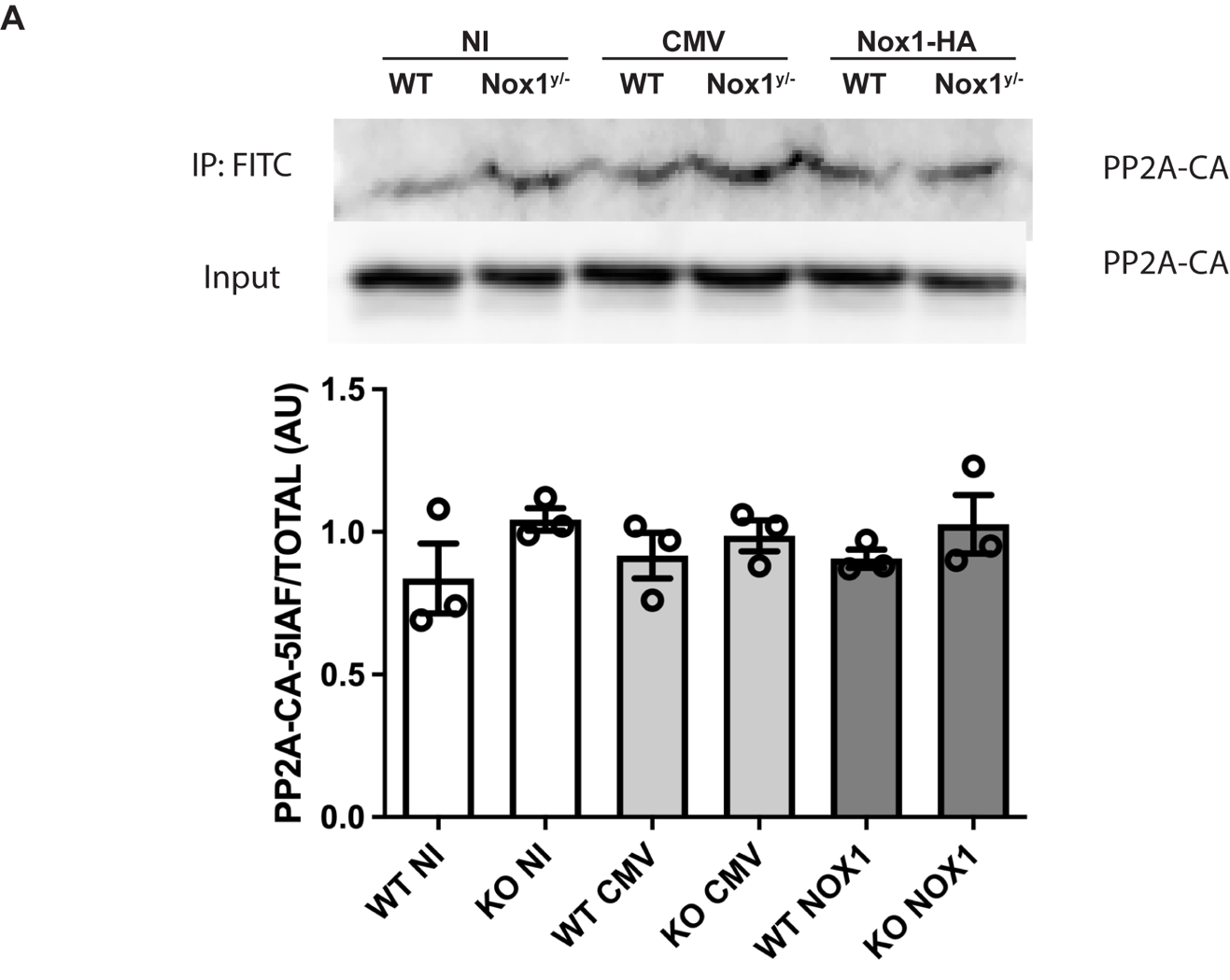


**Supplementary Figure 4. Nox1 expression does not affect PP2A-CA redox status.** WT and Nox1^y/-^ MEFs, were seeded on collagen-I-coated plates and left without infection (NI) or infected with a control virus (CMV) or virus overexpressing Nox-1 (Nox1-HA). After 24 h of infection, cells were serum starved for 16 h to synchronize them and fresh serum containing media was added for 30 min. Cells were lysed in a buffer containing the thiol-reactive probe 5-IAF (5-iodoacetoamidofluorescein), and subsequently precipitated with anti-FITC antibodies. Immunoprecipitated proteins (IP) and total lysates (Input) were separated by Western blot and probed for PPA2-CA. Blots show a representative result out of 3 independent experiments quantified in the graph. Data was analyzed with two-way ANOVA with Tukey correction for multiple comparison. No significant differences were observed.
